# Identification of risk factors and development of a predictive model for chronic kidney disease in patients with obesity: a four-year cohort study

**DOI:** 10.1186/s12944-024-02048-6

**Published:** 2024-02-22

**Authors:** Haixia Zhang, Yue Zhang, Wenxing Gao, Yiming Mu

**Affiliations:** 1 PLA Medical school, Beijing, China; 2 Department of Endocrinology, the First Medical Center of People’s Liberation Army General Hospital, Beijing, China; 3 Department of Endocrinology, Chinese People’s Liberation Army General Hospital, No. 28 Fuxing Road, Beijing, 100853 China

**Keywords:** Prediction model, Chronic kidney disease, Obesity

## Abstract

**Objective:**

The sneaky onset and dismal prognosis of chronic kidney disease (CKD) make it an important public health issue. Obesity-related kidney illness has garnered more attention in recent times. Establishing and validating a risk prediction model for chronic renal illness in overweight or obese adults was the goal of this investigation.

**Methods:**

Data from the China Health and Retirement Longitudinal Study were used for analysis. The definition of CKD was reduced renal function (eGFR < 60 mL/min/1.73 m²), while overweight and obesity were characterized through a body mass index exceeding 24 kg/m². The dataset was divided into derivation and validation cohorts using a 7:3 ratio. With respect to the derivation cohort, we constructed a prediction model using LASSO analysis and multivariate logistic regression. The model’s performance was evaluated using Hosmer-Lemeshow tests, calibration curves, decision curve analysis, and receiver operating characteristic (ROC) curves. The validation cohort’s model was subjected to additional assessment.

**Results:**

The study was based on survey data from 2011 to 2015 and comprised 3246 individuals who were overweight or obese, with 2274 being part of the derivation cohort and 972 being part of the validation cohort. The research constructed a prediction model that included age, sex, fasting blood glucose, glycated hemoglobin, triglyceride, hypertension, and BMI. The validation cohort’s area under the ROC curve was 0.812 (95% CI = 0.763, 0.859) while the derivation cohort’s was 0.789 (95% CI = 0.754, 0.831). Hosmer-Lemeshow tests were utilized to evaluate the model’s accuracy in the validation and derivation cohorts (*P* = 0.681 and 0.547, respectively). The calibration curve showed a high level of consistency between the actual observations and the projected outcomes. According to decision curve analysis, the model offered significant net advantages.

**Conclusions:**

The forecasting model established in this research has predictive value for CKD in patients with overweight or obesity. These findings could help doctors conduct early detection and intervention in clinical practice and further improve patient prognosis.

**Supplementary Information:**

The online version contains supplementary material available at 10.1186/s12944-024-02048-6.

## Introduction

It is anticipated that 13.4% (11.7–15.1%) of people worldwide have chronic kidney disease (CKD), endangering public health [1]. Because of its insidious onset, most CKD patients remain asymptomatic in the early stages, resulting in 90% of cases remaining undiagnosed [2]. As the glomerular filtration rate decreases and albuminuria develops, renal insufficiency progresses, leading to a bleak prognosis, heightened economic burden, and diminished quality of life. Timely detection of declining kidney function has thus become a global focus.

Simultaneously, the surge in global adult obesity rates poses a serious public health challenge. CKD ranks as the second most frequent reason for death among obese individuals [3]. However, renal function test results are not widely available at grassroots community medical institutions, especially in rural areas. Consequently, many obese patients with CKD remain undiagnosed until the advanced stages. Therefore, there is a pressing need to establish a predictive model for CKD by identifying associated risk elements and enabling early identification and intervention to slow or even reverse disease progression.

This study’s objective was to establish the first forecasting model for CKD in people who are overweight or obese in order to identify those who were more likely to experience a deterioration in renal function and would benefit from early intervention.

## Methods

### Study design and participants

**Fig. 1:**
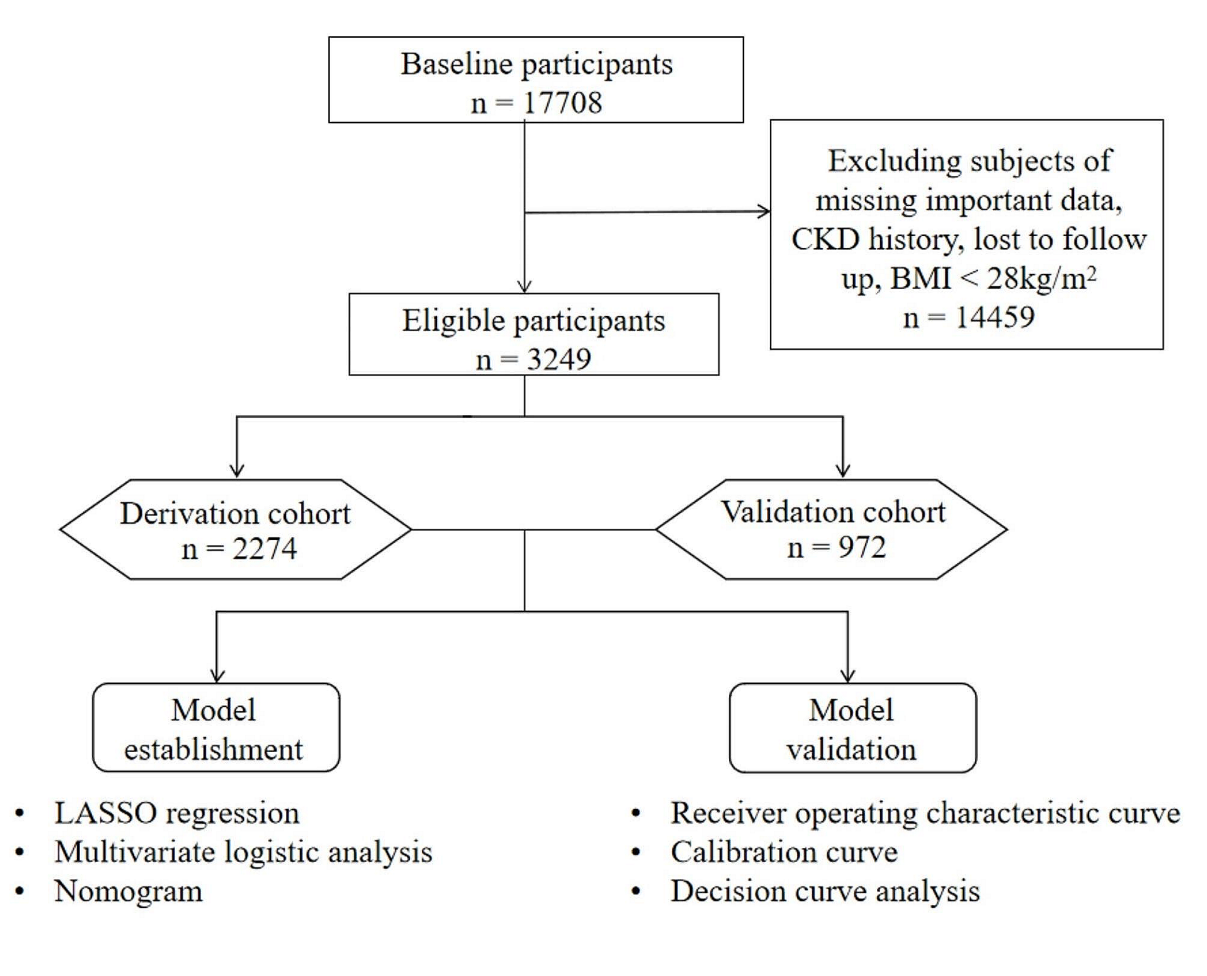
Flow chart of the study

The China Health and Retirement Longitudinal Study provided the study’s data (CHARLS) [4–6], a longitudinal survey conducted in mainland China using multistage stratified random sampling, with a baseline survey starting in 2011, followed by a follow-up survey in 2015. After excluding patients who were lost to follow-up, lacked important data, had CKD at baseline, and had a body mass index (BMI) of less than 24 kg/m^2^ at baseline, 3246 participants were finally included (Fig. 1). Written informed permission was provided by each subject; The Clinical Research Ethics Committee (IRB00001052-11015) at Peking University granted approval for the study, and it was carried out in compliance with the Helsinki Declaration.

### Information collection

Basic information about the participants, including demographic information and lifestyle, disease, and medication history, was collected by trained investigators through standardized questionnaires. Anthropometric data included measures of blood pressure, height, weight, and other parameters. Prior to taking blood pressure readings, each participant was instructed to remain still for a minimum of five minutes. An electronic sphygmomanometer was then utilized to get three readings and then averaged. Venous blood was collected from participants who fasted overnight by trained staff and taken at 4 °C to the local laboratory. In less than two weeks, they were delivered to the Chinese Center for Disease Control and Prevention in Beijing after being frozen at -20 °C. Before being tested at the Capital Medical University laboratory, samples were kept at -80 °C.

### Definition of variable

Weight (kg) divided by height (m^2^) squared was used to compute BMI. Alcohol usage status was classified as either nondrinkers or current drinkers. Smoking status was split into nonsmokers and current smokers. Using the CKD epidemiology (Supplementary table 1), the estimated glomerular filtration rate (eGFR) was computed [7]. If a participant answers “yes” to the question “Have you ever been told that you have kidney disease?” or if an individual’s eGFR less than 60 mL/min/1.73 m^2^, they receive a chronic kidney disease (CKD) diagnosis [8]. A rise in systolic blood pressure of 140 mmHg, a diastolic blood pressure of 90 mmHg, or the use of antihypertensive medications is referred to as hypertension.

### Statistical methods

The Kolmogorov-Smirnov test is used to determine whether the continuous variables are normally distributed. The independent T test as well as the Mann-Whitney U test were applied for comparing the groups’ differences in the continuous variables. Utilizing chi-square analyses, categorical variables were compared and are shown as percentages (%). The prediction model containing the best predictors was first filtered out using ten cross-validations of the least absolute shrinkage and selection operator analysis. Following multivariate logistic regression analysis, independent risk variables linked to the onset of CKD were found. The calibration curve was used to verify that the real results and the predicted model were consistent. By the receiver operating characteristic (ROC) curve, the detection model’s predictive accuracy in the derivation and validation cohorts was assessed. The Hosmer-Lemeshow test was used to determine how well the model fit the data. Both sides *P* < 0.05 is statistically significant. The statistical analysis was performed using R(version 4.0.0) and SPSS (version 22.0).

## Results

### Clinical characteristics of the participants

The study comprised 3246 individuals who were overweight or obese in total. The incidence of CKD was 10.3%. A four-year follow-up period was involved. There were two cohorts created: one for derivation (*n* = 2274) and the other for validation (*n* = 972), with a 7:3 ratio. Table 1 displays the demographic information and clinical data, such as sex, BMI, smoking status and fasting plasma glucose level, of the participants in the two cohorts; these variables did not differ substantially (*P* > 0.05). The features of the derivation cohort’s members according to categories for CKD are displayed in Table 2. The CKD group had substantially higher levels of age, BMI, current smokers, fasting blood glucose, uric acid, total cholesterol, and other indicators in comparison to the normal group (*P* < 0.05). the CKD group had substantially lower levels of eGFR in comparison to the normal group (*P* < 0.05).

**Table 1:** Demographic and clinical characteristics of the derivation and validation cohorts

Variables	Derivation cohort	Validation cohort	*P* value
*n*	2274	972	
Age, years	58.3 ± 8.5	58.1 ± 8.5	0.213
Men, *n* (%)	1046 (46.0%)	448 (46.1%)	0.161
BMI, kg/m^2^	26.6 ± 3.4	26.8 ± 3.4	0.612
SBP, mmHg	130.0 ± 18.5	130.4 ± 18.6	0.558
DBP, mmHg	80.3 ± 10.8	80.5 ± 10.7	0.522
TC, mmol/L	5.0 (4.3, 5.8)	5.0 (4.4, 5.8)	0.245
TG, mmol/L	2.6 (2.1, 3.2)	2.6 (2.1, 3.3)	0.432
HDL, mmol/L	1.3 (1.0, 1.5)	1.3 (1.1, 1.5)	0.347
LDL, mmol/L	3.0 (2.4, 3.6)	3.0 (2.4, 3.6)	0.463
FBG, mmol/L	2.9 (2.5, 3.3)	2.9 (2.5, 3.4)	0.235
HbA1c, %	5.1 (4.8, 5.5)	5.1 (4.7, 5.5)	0.711
C-reactive protein, mg/L	1.0 (0.5, 2.0)	1.0 (0.5, 1.9)	0.322
eGFR, mL/min/1.73m^2^	85.2 (75.4, 96.2)	85.4 (75.6, 96.5)	0.082
Uric acid, mg/dl	4.0 (3.4, 4.7)	4.0 (3.5, 4.7)	0.260
Hemoglobin, g/dl	14.0 (12.9, 15.2)	14.0 (12.9, 15.2)	0.858
Sleep duration, h	7.0 (6.0, 8.0)	7.0 (6.0, 8.0)	0.612
Education status, *n* (%)			0.415
Illiterate	660 (29.0%)	283 (29.1%)	
Primary school and below	952 (41.9%)	405 (41.7%)	
Middle school and above	662 (29.1%)	284 (29.2%)	
Residence, *n* (%)			0.326
Rural	1529 (67.2%)	656 (67.5%)	
Urban	745 (32.8%)	316 (32.5%)	
Marital status, *n* (%)			0.319
Single	245 (10.7%)	106 (10.9%)	
Married/cohabiting	2029 (89.3%)	866 (89.4%)	
Smoking status, *n* (%)			0.256
Non	1978 (87.0%)	845 (86.9%)	
Current	296 (13.0%)	127 (13.1%)	
Drinking status, *n* (%)			0.384
Non	1778 (78.2%)	758 (78.0%)	
Current	496 (21.8%)	214 (22.0%)	

Notes: Data expressed as mean ± standard deviation or median (25th quantile, 75th quantile) for continuous variables and percentage (%) for categorical variables

Abbreviations: BMI: body mass index; SBP: systolic blood pressure; DBP: diastolic blood pressure; TC: total cholesterol; TG: triglyceride; HDL: high-density lipoprotein cholesterol; LDL: low-density lipoprotein cholesterol; FBG: fasting blood glucose; HbA1c: glycosylated hemoglobin; eGFR, estimated glomerular filtration rate

**Table 2:** Demographic and clinical characteristics of derivation cohort by chronic kidney disease category

Variables	Normal group	Chronic kidney disease group	*P* value
*n*	2040	234	
Age, years	58.1 ± 8.5	60.1 ± 8.9	< 0.001
Men, *n* (%)	934 (45.8%)	112 (47.8%)	0.006
BMI, kg/m^2^	26.3 ± 3.3	27.2 ± 3.7	< 0.001
SBP, mmHg	129.0 ± 18.2	133.2 ± 19.0	< 0.001
DBP, mmHg	80.0 ± 10.6	81.9 ± 11.5	< 0.001
TC, mmol/L	4.9 (4.2, 5.7)	5.2 (4.5, 6.0)	< 0.001
TG, mmol/L	2.5 (2.0, 3.1)	2.8 (2.4, 3.5)	< 0.001
HDL, mmol/L	1.3 (1.0, 1.5)	1.2 (1.0, 1.4)	0.001
LDL, mmol/L	2.9 (2.3, 3.5)	3.3 (2.7, 3.9)	< 0.001
FBG, mmol/L	2.8 (2.4, 3.1)	3.4 (2.7, 3.8)	< 0.001
HbA1c, %	5.0 (4.7, 5.4)	5.4 (4.9, 5.9)	< 0.001
C-reactive protein, mg/L	1.0 (0.5, 2.0)	1.0 (0.5, 2.0)	0.127
eGFR, mL/min/1.73m^2^	90.1 (82.9, 105.4)	51.0 (47.5, 56.3)	< 0.001
Uric acid, mg/dl	3.5 (3.1, 4.2)	5.1 (4.5, 5.8)	< 0.001
Hemoglobin, g/dl	14.5 (13.6, 15.4)	13.8 (12.7, 15.0)	0.001
Sleep duration, h	7.0 (6.0, 8.0)	7.0 (6.0, 7.0)	0.023
Education status, *n* (%)			
Illiterate	583 (28.8%)	77 (32.9%)	0.024
Primary school and below	852 (41.8%)	100 (42.7%)	
Middle school and above	605 (29.4%)	57 (24.4%)	
Residence, *n* (%)			0.042
Rural	1367 (67.0%)	162 (69.2%)	
Urban	673 (33.0%)	72 (30.8%)	
Marital status, *n* (%)			0.034
Single	214 (10.5%)	31 (13.2%)	
Married/cohabiting	1826 (89.3%)	203 (86.8%)	
Smoking status, *n* (%)			< 0.001
Non	1779 (87.2%)	199 (85.0%)	
Current	261 (12.8%)	35 (15.0%)	
Drinking status, *n* (%)			0.010
Non	1597 (78.3%)	181 (77.4%)	
Current	443 (21.7%)	53 (22.6%)	

Notes: Data expressed as mean ± standard deviation or median (25th quantile, 75th quantile) for continuous variables and percentage (%) for categorical variables

Abbreviations: BMI: body mass index; SBP: systolic blood pressure; DBP: diastolic blood pressure; TC: total cholesterol; TG: triglyceride; HDL: high-density lipoprotein cholesterol; LDL: low-density lipoprotein cholesterol; FBG: fasting blood glucose; HbA1c: glycosylated hemoglobin; eGFR, estimated glomerular filtration rate

### Construction of the prediction model

**Fig. 2:**
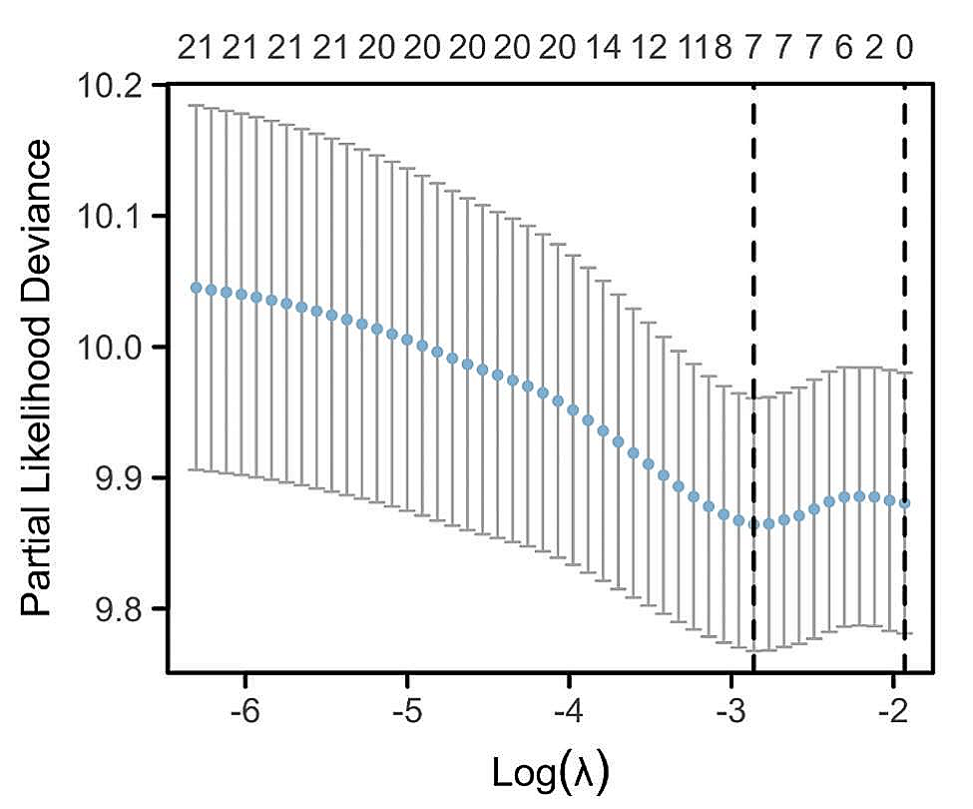
Least absolute shrinkage and selection operator (LASSO) binary logistic regression model. The optimal penalty coefficient λ was identified for the derivation cohort

**Fig. 3:**
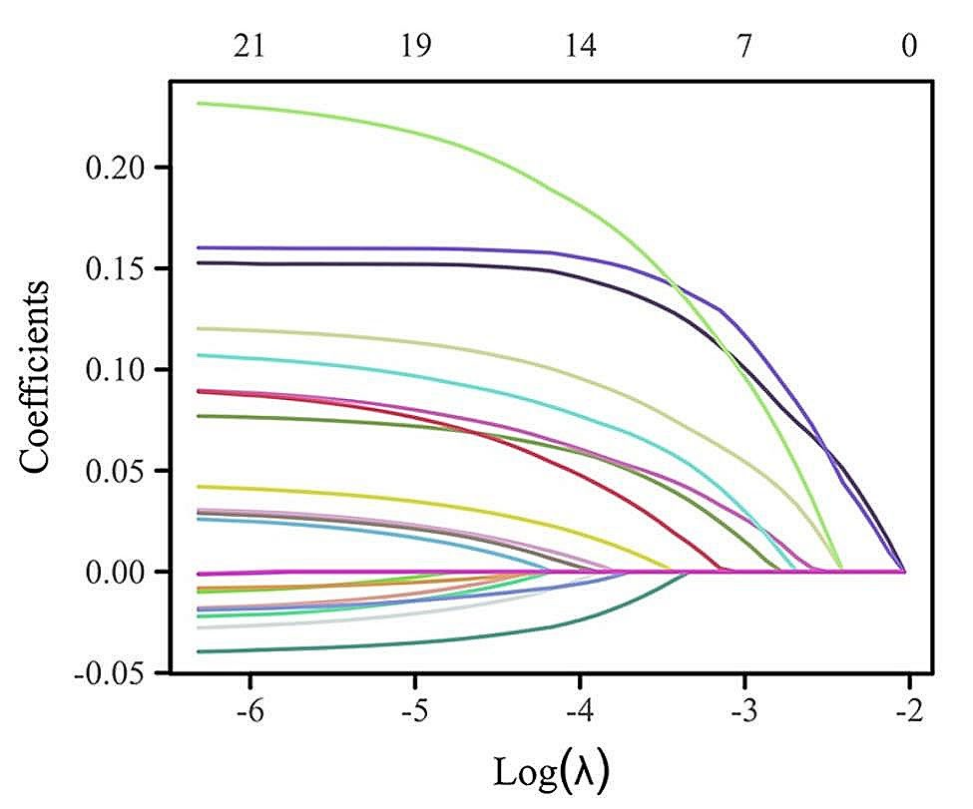
Changes in the LASSO coefficients for the seven variables in the derivation cohort

**Fig. 4:**
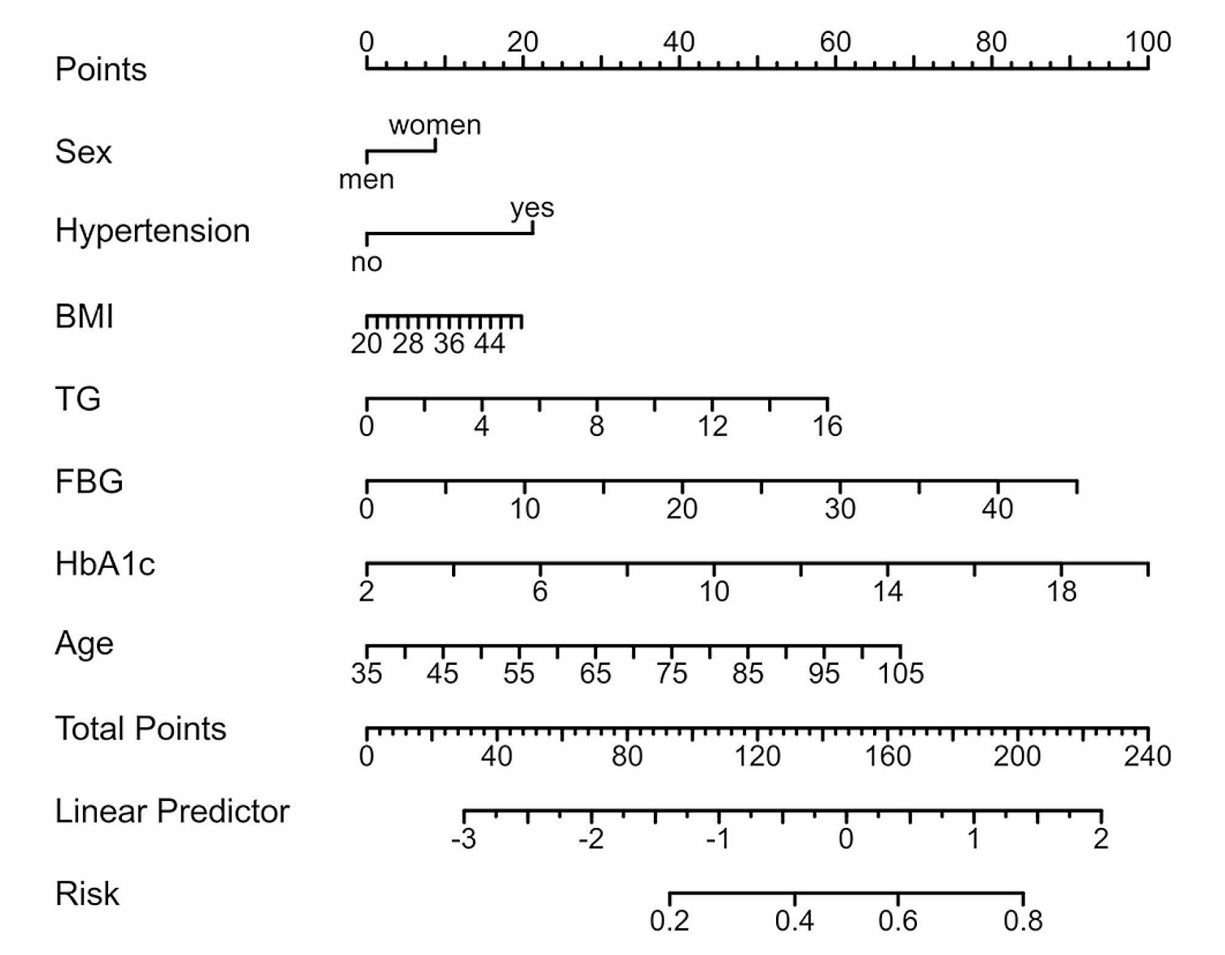
Nomogram for predicting chronic kidney disease in patients with overweight or obesity. Each variable has a separate score, which is summed and then drawn by the vertical line to obtain the total score and the total risk

Based on demographic and clinical data from the derivation cohort, potential predictors were screened by LASSO regression. When seven indicators were included, the best model was reached (Fig. 2). The seven variables, age, sex, fasting plasma glucose, glycated haemoglobin, triglycerides, hypertension, and BMI, had nonzero coefficients in the model (Fig. 3). Multivariate logistic analysis (Table 3) revealed that the associations between these seven factors and CKD were statistically significant and could be considered independent risk factors; therefore, a nomogram was further constructed (Fig. 4). Each risk factor’s individual score was determined using the nomogram’s appropriate scale, and the sum of the scores for each question was used to get the final score. The predicted risk of CKD in patients with overweight or obesity could be obtained by further comparing the percentage at the bottom.

**Table 3:** Multivariate logistic analysis of risk factors to chronic kidney disease in derivation cohort

Variables	Odds ratio	95%CI	*P* value
Age	1.152	1.100-1.208	< 0.001
Sex	1.017	1.001–1.033	< 0.001
HbA1c	1.719	1.549–1.908	< 0.001
FBG	1.251	1.125–1390	0.001
TG	1.099	1.064–1.134	< 0.001
BMI	1.025	1.020–1.031	< 0.001
Hypertension	1.053	1.024–1.083	0.003

Abbreviations: HbA1c: glycosylated hemoglobin; FBG: fasting blood glucose; TG: triglyceride; BMI: body mass index

### Evaluation of predictive models

**Fig. 5:**
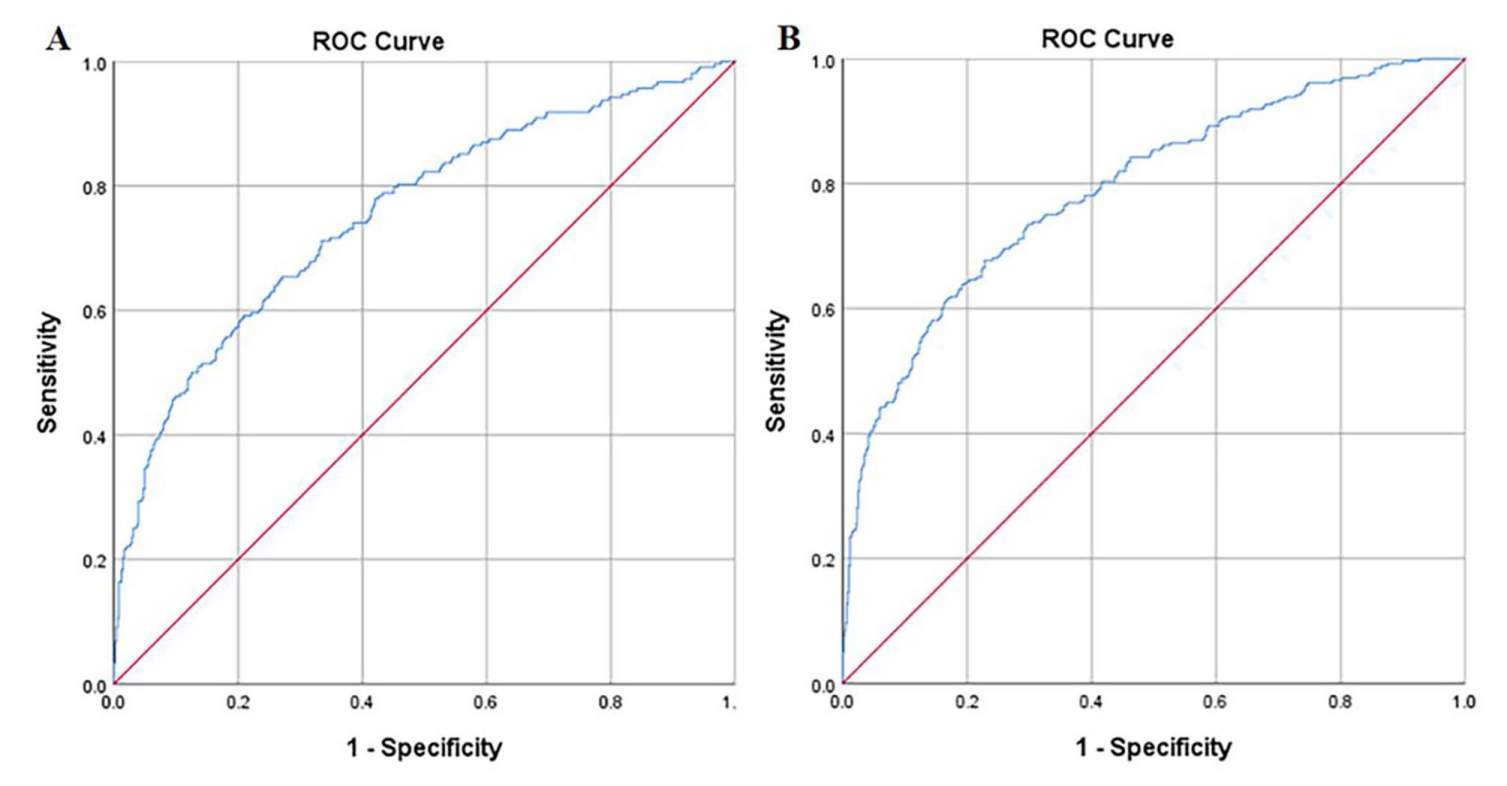
Area under the receiver operating characteristic (ROC) curve of the risk prediction model for chronic kidney disease in patients with obesity in the derivation cohort **(A)** and validation cohort **(B)**

**Fig. 6:**
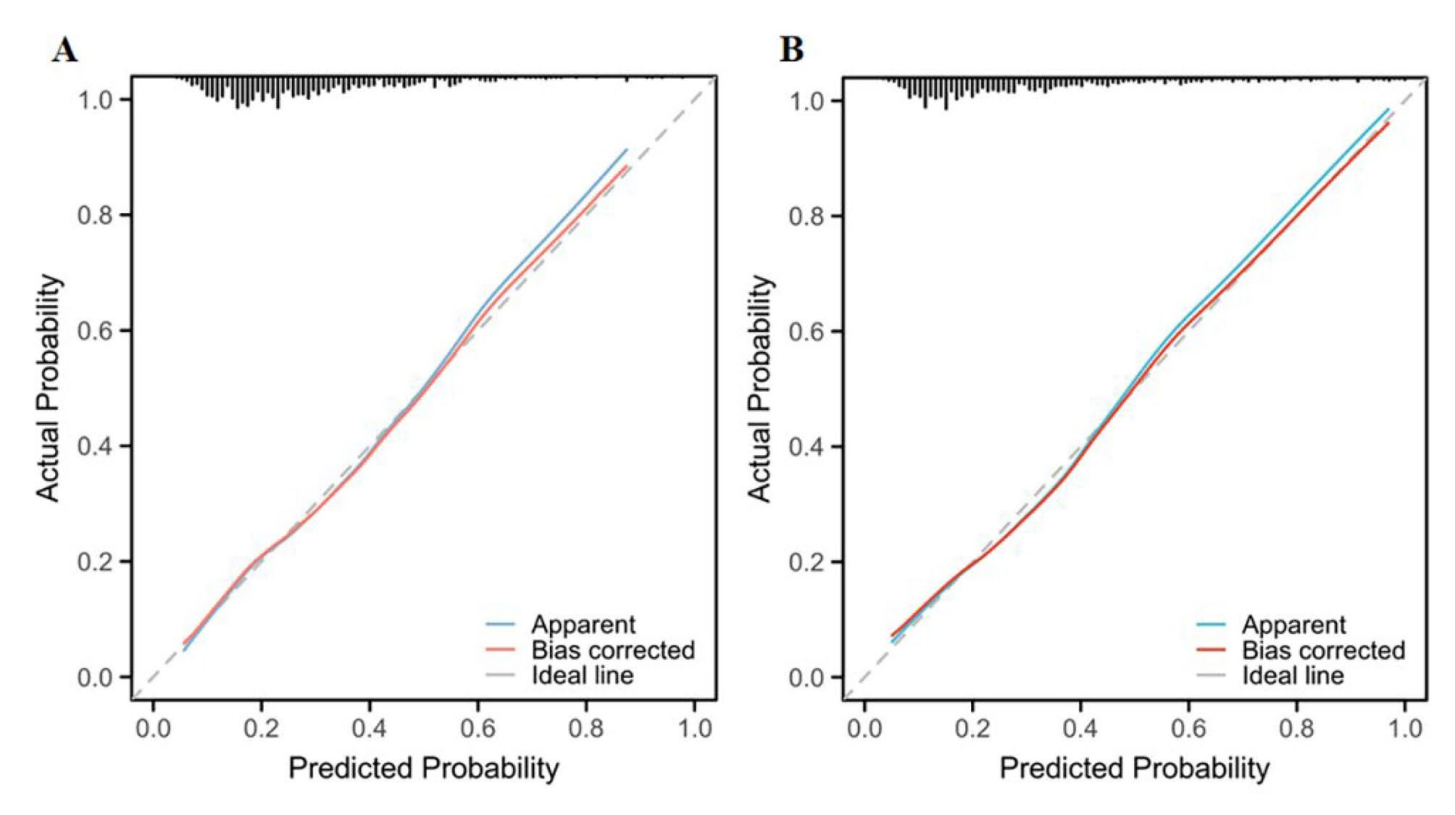
Calibration curves of the nomogram predictions in the derivation cohort **(A)** and validation cohort **(B)**, with diagonal dashed lines representing the ideal state and thin dashed lines representing the actual performance of the nomogram

**Fig. 7:**
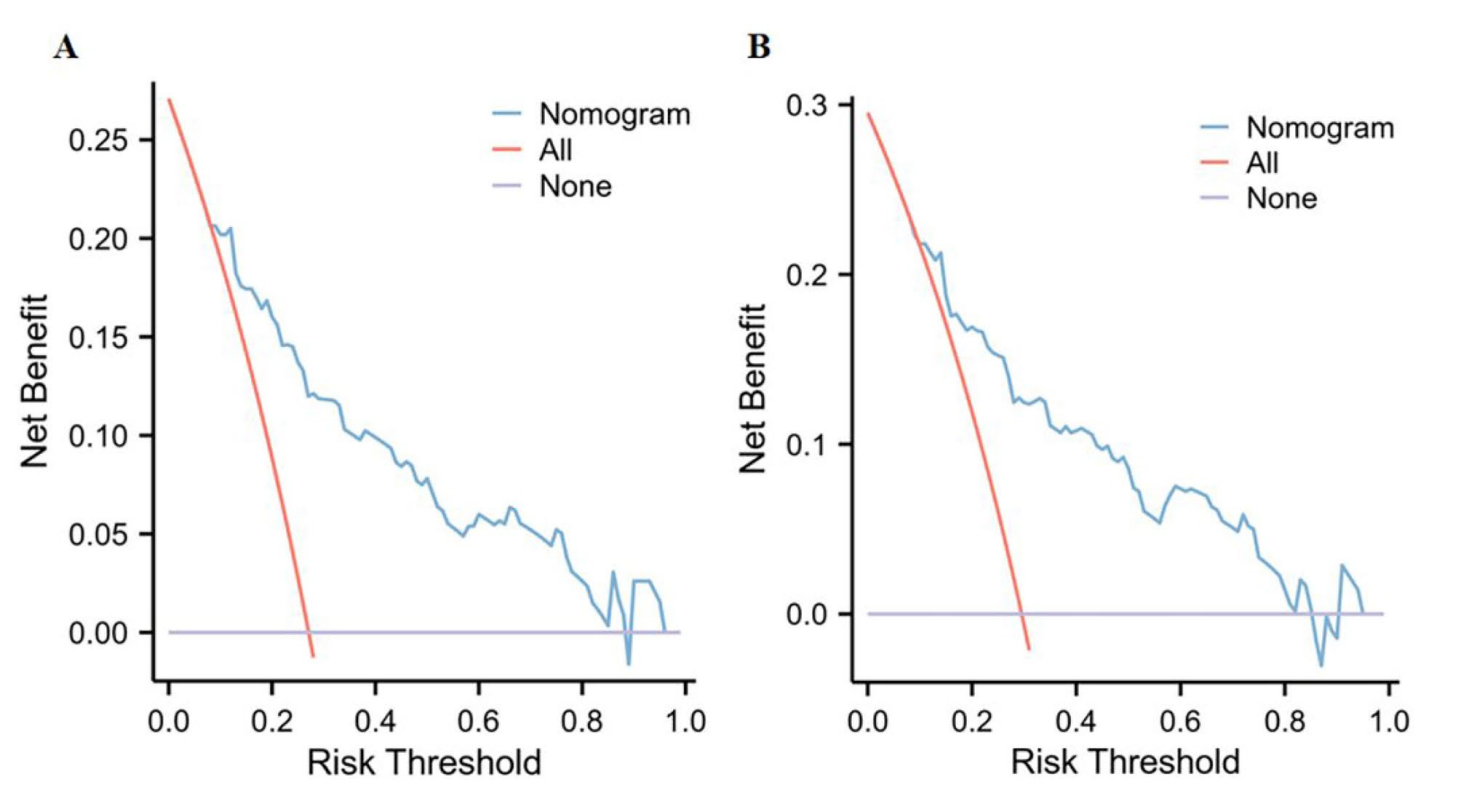
Decision curve analysis of the nomogram predictions in the derivation cohort **(A)** and validation cohort **(B)**

In order to evaluate the nomogram’s clinical value and reliability, ROC curves were produced. In Fig. 5A, the derivation cohort, the area under the ROC curve (AUC) was 0.789 (95% CI: 0.754, 0.831), whereas in Fig. 5B, the validation cohort, it was 0.812 (95% CI: 0.763, 0.859). Additionally, the calibration curves for the validation (Fig. 6B) and derivation (Fig. 6A) cohorts demonstrated a strong correlation between the real data and the nomogram predictions. The study evaluated the model using decision curve analysis (DCA) and discovered that it had high clinical value in the validation (Fig. 7B) and derivation (Fig. 7A) cohorts. The Hosmer-Lemeshow test in the derivation and validation cohorts (*P* = 0.547, *P* = 0.681) was used to evaluate the correctness of the model.

## Discussion

This research concentrated on the risk of CKD among those who are obese or overweight and constructed a predictive column graph that included seven independent risk factors, namely, age, sex, fasting blood glucose, glycated haemoglobin, triglycerides, hypertension and BMI. The calibration chart showed that the projected diagnosis and the actual diagnosis agreed rather well. The results of internal validation were also satisfactory, suggesting that the nomogram has the potential to be used as an efficient and convenient tool for identifying patients with overweight or obesity at high risk of CKD and helping guide clinical decision-making.

Globally, the overweight and obesity rates among adult men and women are as high as 36.9% and 38%, respectively [9], and in China, half of the adult population and one in five children are overweight or obese [10]. Several studies have shown evidence of a correlation between obesity and kidney disease. Being overweight or obese was discovered to be a substantial and potentially adjustable risk factor for the emergence of kidney illness in a large cohort research involving 320,252 adults [11]. A study on Mendelian randomization also demonstrated a causal relationship between high BMI and low eGFR [12].

The incidence rate of CKD is on the rise worldwide. In China, up to 120 million people suffer from CKD [13]. Unfortunately, due to the low awareness rate [14], many patients with CKD enter the dialysis treatment stage immediately after seeing a doctor. Grassroots doctors are the first line of defence for public health. On the one hand, due to the lack of specialists in many primary hospitals, the lack of knowledge and experience in the diagnosis and treatment of CKD directly affects the early diagnosis and treatment of patients with CKD at the primary level; on the other hand, those persons who are overweight or obese, for example, are more likely to experience kidney illness, lack awareness of the long-term standardized management of chronic diseases due to the lack of related risk education. Therefore, the influencing factors of renal function decline have been the focus of worldwide attention. Previous studies and exploration have proved that many independent risk factors related to the development of CKD, such as age [15], gender [13], fasting blood glucose [16], glycated hemoglobin [17], triglyceride [18], hypertension [19], BMI [20], etc., are similar to them. Our studies also have consistent results, but most of these studies are cross-sectional in nature, and the proof of causality is not convincing. This study is the first known cohort study to focus on the risk of chronic kidney disease in obese people.

Given the increasing prevalence of obesity and its occurrence at younger ages, it is imperative to recognize its detrimental impact on multiorgan function throughout the body, the challenges it poses and the financial burden it places on national health care systems. Encouragingly, obesity-related CKD is largely preventable and can be delayed. Research findings indicate that weight loss strategies are beneficial in delaying the advancement of renal damage, resulting in notable improvements in the eGFR and a marked reduction in the rate of urine albumin excretion [21]. Notably, patients with mild CKD and obesity have been found to benefit from a dietary intervention that involves an extremely low-calorie ketogenic diet and has proven to be safe and effective. As a result, up to 27.7% of people with eGFR in the 60-90mL/min/ 1.73m^2^ range have their eGFR levels return to normal after following this dietary intervention [22]. Due to their antiobesity properties, which might affect oxidative stress, insulin resistance, endothelial dysfunction, inflammation, and the advancement of CKD, aerobic exercise and resistance training are advised [23]. Furthermore, bariatric surgery is among the best solutions, not just for losing weight but also for enhancing renal outcomes in overweight or obese individuals with CKD [24]. The renin-angiotensin-aldosterone system (RAAS) inhibitor ramipril has demonstrated notable antialbuminuric effects ,thus lowering the likelihood that obese people may develop end-stage renal disease [25]. Moreover, drugs such as sommarutide (a GLP-1 RA drug) [26] and orlistat [27] have received approval for long-term weight control in obese people, may have a potential kidney protective effect.

During obesity, adipose tissue proximal to the kidneys may induce lipid toxicity, alter adipokine and cytokine secretion patterns, this process leads to podocyte hypertrophy, glomerular enlargement, hemodynamic changes, and fibrosis. Ultimately, this adversely affects nephron function, disrupts the glomerular filtration barrier, and aids in the progression of CKD [24]. Renal impairment is shown to be independently associated with age [14], while sex differences may be associated with lower estrogen levels in postmenopausal women with obesity and increased visceral fat accumulation [28]. Hyperglycemia exacerbates oxidative stress, elevates the production of oxygen-containing free radicals, and fosters the formation of advanced glycation products [29].

Due to the large number of patients with overweight or obesity in China, the incidence of related heart and kidney complications remains high, which also puts great economic pressure on the government. Early screening of Patients at high risk can reduce the overall expense of averting end-stage kidney disease [30]. The advantage of the nomogram is that, on the one hand, it can directly show the relevant factors and the proportion of the outcome events; on the other hand, according to the degree of effect, each value level of each prediction indicator is given a score, which is then added together to get the final score. Ultimately, the functional transformation link between the total score and the likelihood of result events realizes the personalized prediction of the probability of outcome events. This study developed a convenient, accurate, and user-friendly prediction model for CKD, that can help doctors quickly identify high-risk individuals and provide timely lifestyle recommendations and medications to improve patient outcomes.

### Study strengths and limitations

This research has the benefit of being the first to create a CKD prediction model in patients with overweight or obesity. Additionally, this study provides multi-community and large sample data, has been verified internally, and has demonstrated good efficacy. This study also has several limitations. At the stage of data collection, genetic factors and dietary factors, which may have an impact on kidney disease, were not considered. The community residents included were middle-aged and elderly people, which may limit the generalization to other countries and different age groups. Due to the cohort study, loss to follow-up bias is inevitable. The correlation between nonalcoholic fatty liver disease and chronic kidney disease has been confirmed by more and more studies [31, 32]. Unfortunately, NAFLD as a risk factor was not included in this study, which may result in a certain degree of bias.

## Conclusion

The present study aimed to determine the risk variables linked to the beginning of CKD in overweight or obese participants, drawing on the results of a multi-community follow-up investigation. Additionally, a nomogram with strong prediction power was developed. On the one hand, this model provides a visual tool for clinicians to identify high-risk patients and facilitate early intervention to delay disease progression or even reverse the disease; on the other hand, patients can perform self-assessments, improve their living habits, and benefit from these practices.

### Electronic supplementary material

Below is the link to the electronic supplementary material.

Supplementary Table 1

## Data Availability

No datasets were generated or analysed during the current study.

## Abbreviations

CKD: Chronic kidney disease
BMI: Body mass index
SBP: Systolic blood pressure
DBP: Diastolic blood pressure
FBG: Fasting blood glucose
HbA1c: Glycosylated hemoglobin
TC: Total cholesterol
TG: Triglyceride
HDL: High-density lipoprotein cholesterol
LDL: Low-density lipoprotein cholesterol
eGFR: Estimated glomerular filtration rate
ORs: Odds ratios
CI: Confidence intervals
AUC: Area under the receiver operating characteristic curve
