# Supplementary material for: Identification of risk factors and development of a predictive model for chronic kidney disease in patients with obesity: a four-year cohort study
Source: Lipids Health Dis. 2024 Feb 22;23:57. doi: 10.1186/s12944-024-02048-6 (PMC10882765; doi:10.1186/s12944-024-02048-6)
Supplement: Supplementary file 1 — Supplementary Table 1 [file 12944_2024_2048_MOESM1_ESM.docx]

**Supplementary Information**

**Table S1 The CKD-EPI Equation for Estimating GFR.**

| Race and Sex | Serum Creatinine (mg/dL) | Equation |
| --- | --- | --- |
| Black |  |  |
| Female | ≤0.7 | GFR = 166 × (Scr/0.7)^-0.329^ × (0.993)^Age^ |
|  | >0.7 | GFR = 166 × (Scr/0.7)^-1.209^× (0.993)^Age^ |
| Male | ≤0.9 | GFR = 163 × (Scr/0.9)^-0.411^ × (0.993)^Age^ |
|  | >0.9 | GFR = 163 × (Scr/0.9)^-1.209^ × (0.993)^Age^ |
| White or other |  |  |
| Female | ≤0.7 | GFR = 144 × (Scr/0.7)^-0.329^ × (0.993)^Age^ |
|  | >0.7 | GFR = 144 × (Scr/0.7)^-1.209^ × (0.993)^Age^ |
| Male | ≤0.9 | GFR = 141 × (Scr/0.9)^-0.411^ × (0.993)^Age^ |
|  | >0.9 | GFR = 141 × (Scr/0.9)^-1.209^ × (0.993)^Age^ |

CKD-EPI: Chronic Kidney Disease Epidemiology Collaboration; GFR: glomerular filtration rate.
